# Antiproliferative activity of ferulic acid-encapsulated electrospun PLGA/PEO nanofibers against MCF-7 human breast carcinoma cells

**DOI:** 10.1007/s13205-014-0229-6

**Published:** 2014-06-19

**Authors:** Priya Vashisth, Mohit Sharma, Kumar Nikhil, Harmeet Singh, Richa Panwar, Parul A. Pruthi, Vikas Pruthi

**Affiliations:** Department of Biotechnology, Indian Institute of Technology Roorkee, Roorkee, 247667 India

**Keywords:** Antiproliferative activity, Electrospinning, Ferulic acid, Nanofiber, PEO, PLGA, Drug delivery

## Abstract

Ferulic acid (FA) is a polyphenolic phytonutrient which possesses strong antiproliferative effect; however, it has limited therapeutic applications due to its physiochemical instability and low bioavailability at the tumor site. In present study, these shortcomings associated with FA were overcome by fabricating FA-encapsulated poly(d,l-lactide-*co*-glycolide)/polyethylene oxide (PLGA/PEO) blend nanofibers using electrospinning technique. FESEM and fluorescence microscopic analysis imitates the smooth morphology and even distribution of FA within the polymeric nanofibers at optimum 2 wt% concentration of FA. The average diameters were recorded to be 150 ± 47.4 and 200 ± 79 nm for PLGA/PEO and FA-encapsulated PLGA/PEO nanofibers, respectively. The encapsulation, compatibility, and physical state of FA within the nanofibers were further confirmed by FTIR, TGA and XRD analysis. In vitro drug delivery studies demonstrated initial burst liberation of FA within 24 h followed by a sustained release for the subsequent time. MTT assay revealed the effectiveness of FA-encapsulated nanofibers against human breast carcinoma cells (MCF-7) cells as compared to control. FESEM and fluorescence microscopic analysis further confirmed the apoptotic effect of FA-encapsulated PLGA/PEO nanofibers against MCF-7. These fabricated nanofibers hold enormous potential to be used as a therapeutic agent for various biomedical applications.

**Electronic supplementary material:**

The online version of this article (doi:10.1007/s13205-014-0229-6) contains supplementary material, which is available to authorized users.

## Introduction

Phenolic nucleus and unsaturated side chain of FA (4-hydroxy-3-methoxycinnamic acid) readily form a resonance stabilized phenoxy radicals which is responsible for its potent antioxidant capacity. This ubiquitous polyphenolic pharmaceutically active constituent arises from the metabolism of phenylalanine and tyrosine, occurs mainly in seeds, leaves and cell walls of plants such as wheat, rice and oats (Srinivasan et al. 2007). Studies have shown that FA can act as an ingredient of anti-aging supplements, can reduce the level of cholesterol and triglycerides as well as can reduce the risk of cardiovascular dysfunction and inflammatory disease (Ou and Kwok 2004; Murakami et al. 2002). As a multifunctional drug FA exhibits major pharmacological activities such as antioxidant, metal chelation and modulation of enzymatic activities that are responsible for its action to combat deadly infections (Davis and Milner 2010; Ren et al. 2003; Kandaswami et al. 2005). However, despite the extensive potential and beneficiary properties, the therapeutic applications of FA are mostly hampered by its poor solubility in aqueous solution, physiochemical instability, less bioavailability and short half shelf life in the human body fluids (Ouimet et al. 2013; Soobrattee et al. 2005). It has been shown that incorporation of such pharmaceutical active components into polymeric matrix enhanced its bioavailability, hydrophilicity as well as stability (Merlin et al. 2012; Gu et al. 2012). In recent years, biodegradable and biocompatible polymeric nanofibers have been emerged as potential drug carriers. In the same context, Xie and Wang (2006) designed the PLGA-based electrospun fibers for the sustained release of paclitaxel to treat C6 glioma in vitro. Xu et al. (2008) developed doxorubicin hydrochloride containing core–sheath PEG-PLA electrospun nanofibers and studied its release profiles. Electrospinning is a promising method for fabricating ultrafine nanofibers containing anticancer drugs as an effective drug carrier for postoperative local chemotherapy. In a typical electrospinning process, a strong electrostatic field is applied to a polymer solution placed in a syringe with a capillary orifice. When the surface tension of the polymer solution is overcome by the applied electric force, a fiber is extruded from the syringe tip (Huang et al. 2003). This unique technique can produce the fibers with diameters ranging from nanometers to several microns (Vashisth et al. 2013). In this investigation, two biodegradable polymers, PLGA and PEO were used as copolymer blend to fabricate electrospun nanofibers. PLGA is FDA-approved biopolymer and widely used for different therapeutic applications (Vashisth et al. 2013). However, due to its high cost of PLGA polymer, in this study, another biopolymer (PEO) was used along with it, to fabricate cost effective electrospun nanofibers. These fabricated nanofibers were further explored as a drug-carrier for FA. The encapsulation, distribution, physical state and compatibility parameter of these nanofibers were examined. Subsequently, the in vitro release profile of FA from the nanofibers and antitumor activity against MCF-7 breast carcinoma cell lines were also evaluated for confirming their potential therapeutic applications.

## Materials and methods

### Materials

PLGA (average Mw ∼45,000) and PEO (Mw ∼900,000) were purchased from Sigma-Aldrich (St. Louis, MO). Michigan Cancer Foundation-7 (MCF-7) and Human Embryonic Kidney (HEK-293) cell lines were procured from National Center for Cell Science (NCCS), Pune, India. FA, dichloromethane (DCM), *N*,*N*-dimethylformamide (DMF), 3-(4,5-dimethylthiazol-2-yl)-2,5-diphenyltetrazolium bromide (MTT), cell culture-grade dimethyl sulfoxide (DMSO), phosphate buffer saline (PBS), acridine orange (AO), ethidium bromide (EtBr), Dulbecco’s modified Eagle medium (DMEM) and all analytical grade chemicals were from Himedia (India).

### Nanofibers fabrication

PLGA and PEO in a blend ratio of 1:1 were prepared in a solvent mixture of DCM/DMF (4:1, v/v) to prepare a blank electrospinning solution at a final concentration of 2 wt%. FA-encapsulated PLGA/PEO solutions were obtained by dissolving FA at different concentrations (1, 2, 4, 6 and 8 wt%) w.r.t the total polymer concentration in the PLGA/PEO polymeric solution. The FA-free and FA-encapsulated PLGA/PEO solutions were then carefully placed into a 5 mL syringe, attached with a metallic needle (21 G). Electrospinning was carried out under a fixed electric field (15 kV), feeding rate (0.3 mL h^−1^) by means of a single syringe pump (Harvard apparatus 11 plus syringe pumps, US). A piece of aluminum foil was used to collect the nanofibers with the horizontal distance of 12 cm from the needle tip. All electrospinning processes were carried out under ambient conditions (temperature 25 ± 2 °C, relative humidity 60 ± 1 %). The resultant fibers were further dried for 24 h in desiccators to remove the residual organic solvent and moisture.

### Nanofibers characterization

Surface morphology of the nanofibers was observed using field emission scanning electron microscopy (FESEM; Quanta 200F Model, FEI, Netherland) at an accelerated voltage of 15 kV. For sample preparation the nanofibers were cut into rectangular pieces (1 × 1 cm) and sputter coated (sputter coater: Biotech SC005, Switzerland) with gold for 1 min. The average diameters of the nanofibers were measured at over 50 different points of FESEM images (by using image J analyzer software). The morphology of FA-encapsulated PLGA/PEO nanofibers was also confirmed by fluorescent microscopy (Evos fl, AMG groups, USA). The physical state of FA in the electrospun nanofibers was further examined by X-ray diffraction (XRD) analysis. The XRD patterns were recorded with Cu Kα radiation over the 2*θ* range from 5° to 100° with the scanning rate of 2° min^−1^. Thermogravimetric analysis (TGA) of the native FA and nanofibrous samples was performed by using a TGA instrument (EXSTAR, TG/DTA 6300). Each sample (8–10 mg) was kept under vacuum for 24 h prior to testing and then the precisely weighed samples were heated from 23 to 500 °C at a scanning rate of 10 °C min^−1^ under a nitrogen atmosphere. Infrared absorptions of the native FA, PLGA/PEO and FA-encapsulated PLGA/PEO nanofibers were recorded by Fourier transform infrared spectrometer (FTIR; Thermo Nicolet Nexus 6700, US). Scans (16) were recorded in the scanning range of 4,000–500 cm^−1^ with the resolution of 4 cm^−1^.

### Drug encapsulation efficiency

The encapsulation efficiency (EE) of FA in the nanofibers was quantified by thoroughly dissolving the FA-encapsulated nanofibers (containing 2 wt% FA) in a mixture of DCM/DMF (4:1) and the amount of released FA was measured by using UV–Vis spectrophotometer (Lasany double beam LI-2800) at 319 nm. The amount of FA in the fibers was calculated from the obtained data against a predetermined calibration curve for the drug. The encapsulation efficiency of the FA was determined as follows:

EE (%) = Actual FA content in nanofibers (mg)/Theoretical FA content in nanofibers (mg) × 100.

### In vitro drug release

In vitro release studies of FA from the 2 wt% FA-encapsulated PLGA/PEO nanofibers were performed by incubating 10 mg nanofibrous mat in 30 mL PBS (pH 7.4). The incubated samples were maintained in a thermostat (37 °C) at 50 rpm. At predetermined time intervals, 3 ml of samples was withdrawn from dissolution medium and the OD was measured at the 319 nm using UV/Vis spectrophotometry.

### In vitro cytotoxicity

In vitro cytotoxicity of the PLGA/PEO and 2 wt% FA-encapsulated PLGA/PEO nanofibers toward MCF-7 cells was evaluated using MTT assay (Mosmann 1983). Briefly, the UV sterilized nanofibrous samples were first placed in 24-well microtiter plates individually followed by the addition of MCF-7 cells (5 × 10^5^ cells mL^−1^) along with DMEM medium supplemented with 10 % fetal bovine serum (FBS). The plates were then incubated in humidified atmosphere containing 5 % CO_2_ at 37 °C for 24 h. MCF-7 cells grown in presence of culture medium only were used as negative control, whereas the MCF-7 cells cultured in presence of native FA only were used as a positive control. Subsequently, the MTT solution (60 μL of 5 mg mL^−1^ stock) was added to each well containing sample, and incubated at 37 °C for 4 h. Then, the solution in the wells was removed carefully and 400 μL of DMSO was added to dissolve the MTT formazan crystals. After that, 100 μL of the dissolved formazan solution of each test sample was transferred to individual wells of 96-well plate to determine the absorbance at 570 nm (*A*_570nm_) using micro-plate reader (Fluostar optima, BMG labtech, Germany). The cell viability was calculated as follows:

$$\text{Cell viability}\;\left(\%\right)\;={\text{OD}}_{570\text{nm}}\left(\text{test samples}\right)/{\text{OD}}_{570\text{nm}}\left(\text{control}\right)\times \;100$$

Analysis of change in morphology of MCF-7 tumor cells in presence of native FA and FA-encapsulated nanofibers.

The UV-sterilized nanofibrous samples were placed in 24-well plates, seeded with MCF-7 cells (5 × 10^5^ cells mL^−1^) and incubated at 37 °C for 24 h in a CO_2_ incubator. The samples were then washed thrice with PBS (pH 7.4) immediately after the incubation period to remove any unattached MCF-7 cells. The cell morphologies for each sample were first observed using an inverted phase contrast microscope (Zeiss, Axiovert 25, Germany) at 100× magnification. For FESEM examination, the MCF-7 tumor cells adhered to nanofibers were fixed by immersing the samples into 2.5 % glutaraldehyde PBS solution at 4 °C for 4 h. Subsequently, the samples were dehydrated stepwise with graded concentrations of ethanol (25, 50, 75, 90 and 100 %).

### Analysis of cell death using fluorescent microscopy

MCF-7 cells (5 × 10^5^ cells mL^−1^) cultured on the different formulations (control, free FA, PLGA/PEO nanofibers and FA-encapsulated PLGA/PEO nanofibers), as described above were also visualized by double staining with acridine orange (AO) and ethidium bromide (EtBr). Briefly, after 24 h of incubation, the MCF-7 tumor cells seeded samples were rinsed thrice with PBS and instantly stained with equal volumes of AO and EtBr (100 µg mL^−1^ stock solution) for 10 min. The samples were then visualized under the fluorescence microscope (Evos fl, AMG groups, USA).

### Cytocompatibility evaluation

The cytocompatibility of different formulation (native FA, PLGA/PEO and FA-encapsulated PLGA/PEO nanofibers) was evaluated using HEK-293 cells. Briefly, the sterilized formulations were first placed individually in 24-well microtiter plates followed by addition of HEK-293 cells (5 × 10^4^ cells mL^−1^), seeded in DMEM medium supplemented with 10 % FBS, 100 U mL^−1^ penicillin, and 100 µg mL^−1^ streptomycin. The plates were then incubated in a humidified atmosphere with 5 % CO_2_ at 37 °C for 24 h. Coverslips without nanofibers were used as controls. After 24 h of cell seeding, MTT assay (*A*_570nm_) and FESEM studies were performed as described above.

### Statistical analysis

Each measurement was performed at least in three independent experiments (*n* = 3) and the data are expressed as the mean values with ±standard deviation (SD). Statistical analysis of the differences between mean values obtained from experimental groups was performed using one-way ANOVA test. Values of *p* < 0.05 or less were considered as denoting statistical significance.

## Results and discussion

### Morphology of FA-free and FA-encapsulated PLGA/PEO nanofibers

**Fig. 1:**
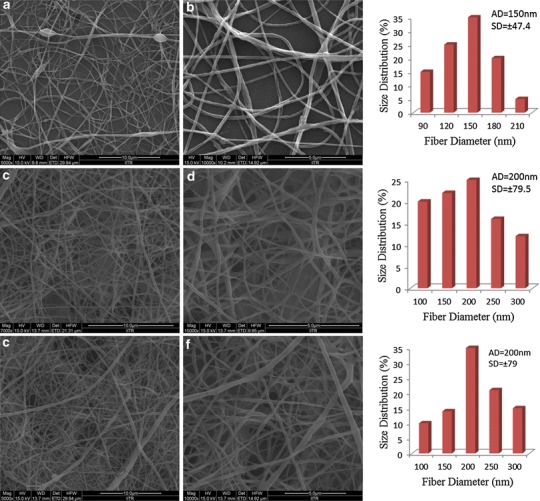
FESEM micrographs and corresponding diameter distribution histogram of 2 wt% PLGA/PEO nanofibers (**a**, **b**) 1 wt% FA-encapsulated PLGA/PEO nanofibers (**c**, **d**) and 2 wt% FA-encapsulated PLGA/PEO nanofibers (**e**, **f**); *Scale bar* (**a**, **c**, **e**) = 10 μm and (**b**, **d**, **f**) = 5 μm. *SD* standard deviation, *FESEM* field emission scanning electron microscope, *AD* average diameter; *n* = 5

The key processing parameters that influence the formation as well as the morphology of electrospun nanofibers include concentration of electrospinning solution, surface tension, nature of the solvent used, applied voltage, solution flow rate, and the distance between tip and collector (Zhang et al. 2012). In this study, all the above described parameters were optimized for obtaining the nanofibers with desired morphology and FA distribution. The FESEM micrographs of the PLGA/PEO nanofibers and their average diameter have been presented graphically in Fig. 1. Data obtained from FESEM characterization demonstrated the beads free uniform morphology of PLGA/PEO nanofibers. The surface morphology of FA-encapsulated PLGA/PEO nanofibers at 1 and 2 wt% concentration of FA was found to be cylindrical, smooth, and free of any bead defect but the presence of FA caused entanglement in the fabricated PLGA/PEO nanofibers. However, no FA crystals were noticed on encapsulated nanofibrous surface which suggests the homogenous dispersion of FA within the PLGA/PEO nanofibers (Fig. 1c–f). Data also depicted the increase in average fiber diameter after FA-encapsulation and recorded as 150 ± 47.4 to 200 ± 79 nm for PLGA/PEO and FA-encapsulated PLGA/PEO nanofibers, respectively (as presented in corresponding histograms in Fig. 1).

**Fig. 2:**
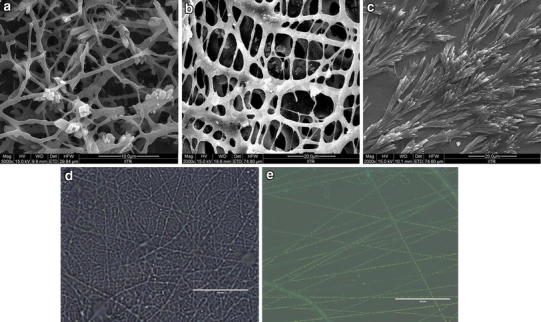
FESEM micrographs of 4, 6 and 8 wt% FA-encapsulated PLGA/PEO nanofibers (**a**, **b**, **c**), respectively and overlay fluorescent micrographs of PLGA/PEO and 2 wt% FA-encapsulated PLGA/PEO nanofibers (**d**, **e**), respectively (*bar* 50 μm)

At higher FA concentration (4 wt%) the surface morphology of nanofibers changed from smooth, cylindrical, defect free to rough and damaged surfaces (Fig. 2a). Some drug crystals and several agglomerates were also observed at the exterior surface of the nanofiber. On further increasing concentrations of FA (6 and 8 wt%) instead of fiber formation, electrospraying of drug-polymeric solution was observed (Fig. 2b, c) (Almería et al. 2010). These observations suggest that the higher concentration of FA does not support electrospinning process and disrupt the morphology as well as uniformity of the electrospun nanofibers. Further, the autofluorescence properties of FA were exploited for visualizing the distribution of it within the PLGA/PEO nanofibers. The overlay fluorescent images of FA-encapsulated (containing 2 wt% FA) PLGA/PEO nanofibers as shown in Fig. 2d and e, represent the core-sheath arrangement of FA within the PLGA/PEO fibers. These observations suggested that the 2 wt% FA is the optimum/maximum amount of drug that can be encapsulated in the above fabricated nanofibers. Furthermore, it seemed that incorporation of the FA at lower concentration in the PLGA/PEO nanofibers did not affect the morphology of the resulting fibers.

### Analysis of physical state of FA within PLGA/PEO nanofibers

**Fig. 3:**
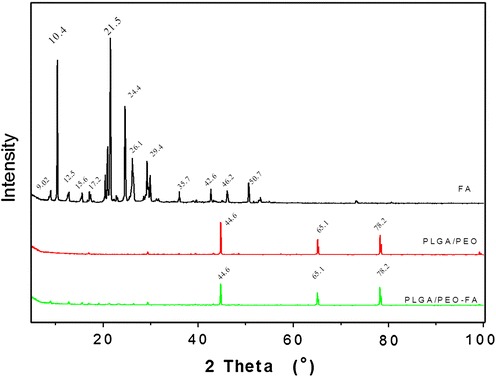
X-ray diffraction patterns of the native FA and nanofibrous formulations

Native FA, PLGA/PEO nanofibers and 2 wt% FA-encapsulated PLGA/PEO nanofibers were examined through XRD technique in order to reveal the physical state and distribution of the drug (FA) in the electrospun nanofibers. As shown in Fig. 3, FA exhibits several characteristic peaks at 2*θ* = 9.02, 10.4, 12.5, 15.6, 17.2, 24.4, 29.4, 35.7, 42.8, 45.8, and 50.7 (Yu et al. 2013). The XRD patterns of FA-free PLGA/PEO nanofibers exhibit typical crystalline peaks at 2*θ* = 44.6, 65.1 and 78.2. The composite FA-encapsulated PLGA/PEO nanofibers possessed the same peaks at 2*θ* = 44.6, 65.1 and 78.2 as that of PLGA/PEO nanofibers. Observations revealed that loading of the FA within the nanofibers did not change the typical crystalline nature of the PLGA/PEO nanofibers. The peaks that attributed to crystalline FA disappeared in the XRD spectrum of composite PLGA/PEO-FA nanofibers confirmed the amorphous state of FA in the composite nanofibers.

**Fig. 4:**
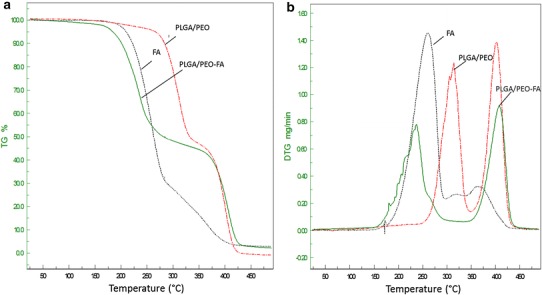
TGA (**a**) and DTG (**b**) thermograms of the native FA and nanofibrous formulations

Thermal properties of the native FA, PLGA/PEO, and FA-encapsulated PLGA/PEO nanofibers were analyzed by TGA as shown in Fig. 4a. FA exhibits a single stage thermal degradation with decomposition starts around 173 °C which is similar to the studies of Mathew and Abraham (2008). However, the decomposition of PLGA/PEO and FA-encapsulated PLGA/PEO nanofibers was recorded in two stages. The initial weight loss in the range of 50–200 °C was due to the evaporation of physically weak and chemically strong H_2_O bonding. The second stage of decomposition started at around 260 °C and completed at around 400 °C. This zone was found to be the highest thermal degradation zone which corresponds to a complex process including depolymerization as well as decomposition of bonds and units of polymers (Fouad and Elsarnagawy 2013; Ibrahim and Johan 2012). The result obtained suggested that there was no significant difference in the thermal stability of PLGA/PEO and FA-encapsulated PLGA/PEO nanofibers.

The derivative TGA curves of weight loss for native FA, PLGA/PEO and FA-encapsulated PLAG/PEO nanofibers are shown in Fig. 4b. Each temperature peak in the thermograms represents the *T*_max_, corresponds to the maximum degradation rate. FA exhibits a peak of maximum degradation at 263 °C while PLGA/PEO nanofibers exhibited two peaks at 315 and 402 °C. However, the peaks of maximum decomposition for FA-encapsulated PLGA/PEO nanofibers were observed at 240 and 408 °C. The slight shifting of temperature peak toward the lower temperature was perceived in case of FA-encapsulated PLGA/PEO nanofibers as compared to native FA. This shifting indicated the presence of weak interactions between polymer components (PLGA/PEO) and FA as well as reflected the early onset of FA decomposition in nanofibers than that of native FA. This may be due to the even distributions and presence of amorphous state of FA in PLGA/PEO nanofibers which make FA molecules respond to the heat more sensitively than native FA crystalline particles.

### Analysis of secondary interactions among the components

**Fig. 5:**
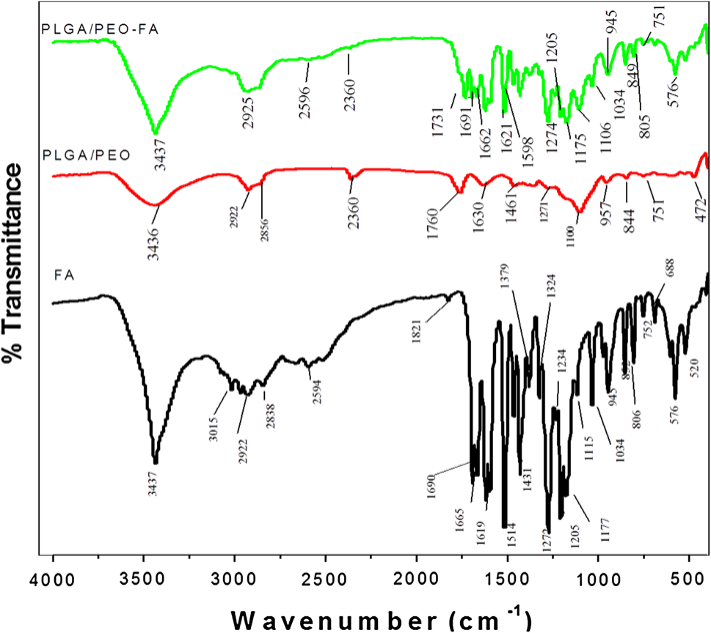
FTIR analysis of the native FA and nanofibrous formulations

**Fig. 6:**
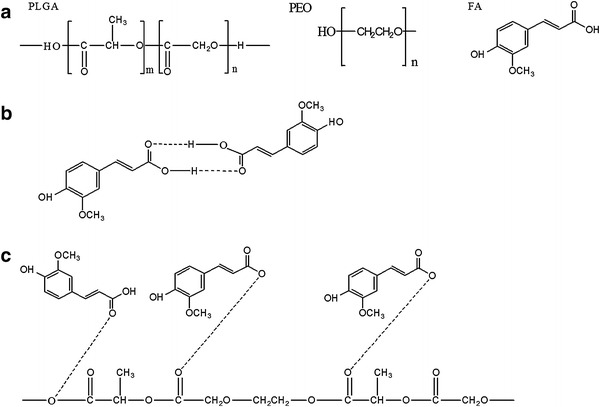
**a** Molecular structures of PLGA, PEO and FA. **b** Hydrogen bonding between FA molecules. **c** Interaction between FA and PLGA-PEO molecules

Secondary interactions in terms of hydrogen bonding, hydrophobic interactions, and electrostatic forces are often considered to be true indication of compatibility between the polymer and drug component for producing high quality, stable nanofiber. As shown in Fig. 5, FA molecule has both –OH and –C=O active groups and exhibited characteristic absorption bonds at 3,437 cm^−1^ (–OH group), 3,015, 2,922, 2,838, and 2,594 cm ^−1^ (C–H bond stretching), 1,690, 1,665, and 1,619 cm ^−1^ (–C=O stretching), 1,272–1,034 cm ^−1^ (–C–O stretching) as also reported earlier by Yang et al. (2013) and Yu et al. (2010). The PLGA/PEO nanofibers showed the absorption peaks at 3,436 cm ^−1^ (–OH stretching), 2,922 and 2,856 cm ^−1^ (–CH stretching), 2,360 cm^−1^ (C≡C stretching), 1,760 and 1,630 cm^−1^ (–C=O stretching), 1,271 and 1,100 cm ^−1^ (C–O stretching). All the entire sharp peaks for FA can be observed in the IR spectrum of FA-encapsulated PLGA/PEO-FA nanofibers, indicating that FA in the composite nanofibers more likely tends to form FA dimers that are necessary for constructing a crystal lattice. The shift of 1,640–1,820 cm ^−1^ absorption bands to lower wave numbers in the spectrum of FA-encapsulated PLGA/PEO nanofibers illustrated the presence of weak interactions between the carbonyl groups of PLGA/PEO blend and the hydroxyl group of FA (Fig. 6). These weak interactions between the FA and PLGA/PEO nanofibers imitated the compatibility between the FA and nanofibers.

### In vitro drug-release assay

**Fig. 7:**
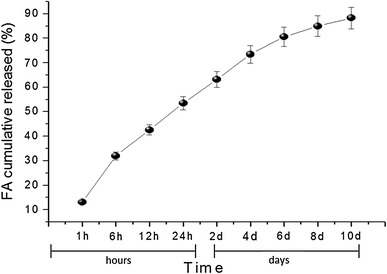
Invitro drug-release profile of FA from the FA-encapsulated PLGA/PEO nanofibers at different time intervals

The amount of drug-loading and the drug-release characteristic can be controlled by investigating the actual drug content in the resulted fibers (Han et al. 2009). The EE of FA in the 2 wt% FA-encapsulated PLGA/PEO nanofibers was recorded as 66 ± 1.34 %. The sustainability of FA molecules in the PLGA/PEO nanofibers was investigated by in vitro drug-release profiles in physiological condition at different time points. The release rate of encapsulated FA revealed an initial burst release within the 24 h, followed by a continuous and sustained release during the subsequent time (Fig. 7). More than half (approx. 278 µg, 53.51 %) of the encapsulated FA was released from the nanofibers within 24 h of incubation. At the end of the 240 h time interval, 89 % (approx. 459.13 µg) of FA had been found to be released from the PLGA/PEO nanofibrous matrix. The initial burst release of FA is possibly due to the dissolution/ or diffusion of FA molecules that are entrapped/ or adsorbed at the surface of polymeric nanofibers. However, the sustained release of FA in subsequent stage may be attributed either to the diffusion of encapsulated-FA from the core region of nanofibers or due to degradation of polymeric matrix. The results obtained are also supported by the findings of Merlin et al. (2012) and indicated that these nanofibers can be potentially useful for sustained delivery of drug for cancer treatment (Cui et al. 2010).

### In vitro cytotoxicity

**Fig. 8:**
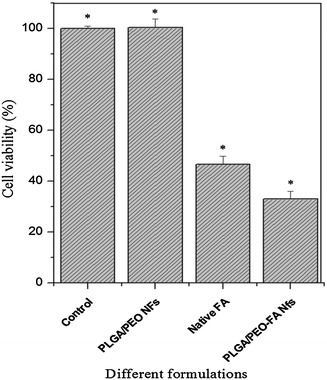
Colorimetric MTT viability assay for MCF-7 cells treated with different formulations coverslips (control), PLGA/PEO nanofibers, native FA (in solution form) and FA-encapsulated PLGA/PEO nanofibers after 24 h at 570 nm. *Error bars* represent mean ± standard deviation for three independent experiments (*n* = 3). *p < 0.05 indicates statistical significant difference as compared to control. (*Nfs* nanofibers)

Globally, breast cancer has become one of the most frequently diagnosed cancer and second leading causes of female malignancy in India (Indap and Radhika 2006). In spite of huge progress in cancer treatments, the assimilated or inherent resistance of breast cancer cells toward the anticancer agents is the prime hurdle for effective chemotherapeutic treatments. Hence, the search for new and effective chemotherapeutic agents is the requisite for treatment of drug-resistant breast cancers (Ignatova et al. 2011). FA is a therapeutically renowned phytochemical which has been reported to exhibit antitumor activities against colon cancer (Kawabata et al. 2000), skin tumor (Asanoma et al. 1994), and pulmonary cancer (Lesca 1983). The antiproliferative effect of FA is mainly attributed to its inhibitory effect toward formation of nitrosamine in the cells (Kuenzig et al. 1984). Till now, no report regarding to the antitumor activity of nanofibers containing FA against MCF-7 has been reported. Therefore, in present investigation, in vitro cytotoxic effects of the FA-encapsulated nanofibers toward MCF-7 cells have been evaluated. As shown in Fig. 8, the 2 wt% FA-encapsulated PLGA/PEO nanofibers possess a strong antiproliferative activity toward MCF-7 cells as compared to control. No inhibition in cell growth was recorded in control and PLGA/PEO nanofibrous sample. However, in case of native FA and FA-encapsulated PLGA/PEO nanofibers, the cell inhibition rate of 51.4 % and 67 % (*p* < 0.01) was achieved, respectively. Moreover, it could be interpret from the results that FA retains its activity even after encapsulation into nanofibrous matrix and is not affected by the electrospinning process. The polymeric nanofibers performed as preservative agent for FA which improved its physiochemical stability as well as protected it from the premature degradation (Supplementary Fig. 1). Therefore, the encapsulation of FA in PLGA/PEO nanofibers resulted into enhanced activity of encapsulated FA in comparison to native FA in solution form. Similar studies have also been reported by Ouimet et al. (2013) on FA-containing polyanhydride esters.

### Morphological analysis of MCF-7 cells in presence of FA-encapsulated nanofibers

**Fig. 9:**
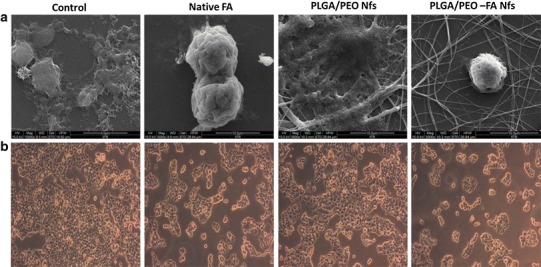
The effect of different formulations (control, native FA, PLGA/PEO nanofibers and FA-encapsulated PLGA/PEO nanofibers) on cell morphology of MCF-7 cells **a** FESEM and **b** phase contrast micrographs of the MCF-7 cells after 24 h of treatment with different formulations

The change in cell morphologies of the MCF-7 cell in presence of native FA and FA-encapsulated PLGA/PEO nanofibers is illustrated in Fig. 9. Tumor cells cultured on PLGA/PEO nanofibers did not comprise any changes in their morphology as well as in the cell growth (Fig. 9a, c). In contrast, the MCF-7 tumor cells possessed good adhesion and proliferation properties onto the PLGA/PEO nanofibers. A great number of tumor cells that adhered to PLGA/PEO nanofibers were found to acquire normal bilateral symmetric morphology and also displayed numerous microvilli on their surface, similar to control.

The FESEM images depicted the significant reduction in tumor cell number/growth when cultured on FA-encapsulated nanofibers. The cell blebbings on the tumor cells surface and disappearance/reduction of the microvilli were also observed in presence of native FA solution and FA-encapsulated nanofibers (Fig. 9d). Our observations are found to be in agreement with the findings of Ignatova et al. (2011).

### Analysis of cell death using fluorescent microscopy

**Fig. 10:**
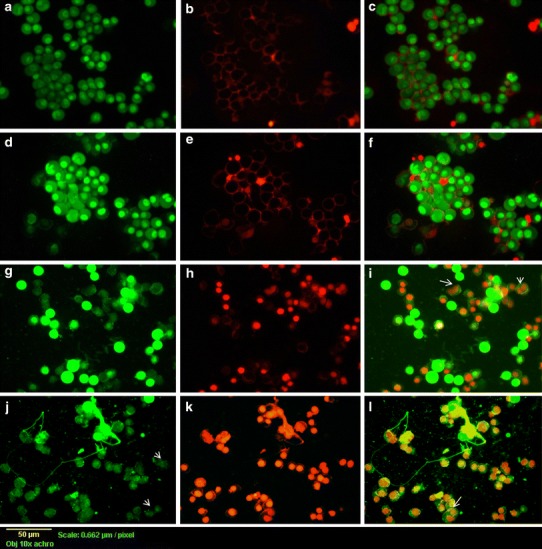
Fluorescent micrographs of AO:EtBr stained MCF-7 cells incubated for 24 h **a**–**c** untreated cells, **d**–**f** cells treated with PLGA/PEO nanofibers, **g**–**i** treated with native FA and **j**–**l** cells treated with FA-encapsulated PLGA/PEO nanofibers. The significance of *arrows* is elucidated in text

To determine the cell cytotoxicity, double staining of MCF-7 tumor cells cultured in the presence of different formulations (control, native FA, PLGA/PEO nanofibers and FA-encapsulated PLGA/PEO nanofibers) was executed using equivalent mixture of AO and EtBr (1:1 w/w). The stained cells on different formulations were then analyzed by fluorescent microscopy. AO and EtBr stain DNA, which allows visualization and differentiation of dead and viable cells. Cells with intact membranes stained green due to passage of AO whereas EtBr stained the cells with damaged membranes, as a result, the DNA intercalation of both gives orange fluorescence (Fig. 10). No cell death was observed in control MCF-7 cells (Fig. 10a–c). Similarly, no growth inhibition was recorded in case of FA-free PLGA/PEO nanofibers (Fig. 10d–f) whereas a considerable amount of damaged MCF-7 cells were get into notice when exposed to native FA (94.3 %, *p* < 0.05) and FA-encapsulated nanofibers (99.8 %, *p* < 0.05). The cells cultured on native FA (Fig. 10g–i) and the FA-encapsulated nanofibers (Fig. 10j–l) displayed the morphological signs for apoptosis such as cytoplasmic remnants, chromatin condensation and damaged wrinkled cells with orange fluorescence (as specified by arrows), indicated that induction of apoptosis is one of the major mechanism through which FA exerts its action to destroy tumor cells. The significant amount of apoptotic cells after treatment of FA-encapsulated PLGA/PEO nanofibers indicated that the encapsulated FA would be able to induce apoptosis more efficiently and possessed a substantial strong antitumor capacity as compared to native FA. These observations are in line with the work done by earlier researchers, who described the anti-apoptotic role of FA on different cancer cell lines (Khanduja et al. 2006; Chen et al. 2007; Indap and Radhika 2006).

### Cytocompatibility assay

**Fig. 11:**
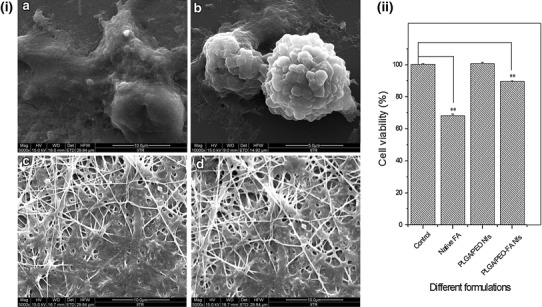
**i** FESEM micrographs of the HEK-293 cells incubated for 24 h on *a* control, *b* in presence of native FA, *c* PLGA/PEO nanofibers and *d* FA-encapsulated PLGA/PEO nanofibers. **ii** MTT assay for HEK-293 cells treated with different formulations for 24 h (*A*_570nm_). *Error bars* represent mean ± standard deviation for three independent experiments (*n* = 3). **p < 0.01 indicated statistical significant difference and (*) *unmarked bar* indicated statistically insignificant difference as compared to control

To evaluate the prospective biomedical applications of these FA-encapsulated PLGA/PEO nanofibers, cytocompatibility assay was carried out against HEK-293 cells using MTT assay. The viability of HEK-293 cells seeded on different formulation is shown in Fig. 11. Based on the results, it was observed that the viability of cells on PLGA/PEO and FA-encapsulated PLGA/PEO nanofibers was much higher than in presence of native FA, indicating the better cytocompatibility of FA-encapsulated nanofibers in comparison with native FA (*p* < 0.01). Data obtained, imply that the incorporation of FA within the nanofibers did not compromise the cytocompatibility of PLGA/PEO nanofibers. The cytocompatibility evaluation of FA-encapsulated PLGA/PEO nanofibers as compared to the native FA, was also performed via cell surface morphology observations (Fig. 11i). The cell adherence, proliferation and migration of HEK-293 cells cultured on both type of nanofibers was found to be similar as on native extracellular matrix. However, the blebbings on cell surface were observed in case of cells cultured in presence of native FA. The cell viability was found to be lower on FA-encapsulated nanofibers as compare to PLGA/PEO nanofibers which may be attributed to the antioxidant property of FA (Srinivasan et al. 2007).

## Conclusion

FA, a potent phytonutrient, was successfully encapsulated in the blend PLGA/PEO nanofibers using electrospinning technique to improve both stability as well as efficiency of FA. Microscopic studies revealed the homogenous distribution of FA as a core-sheath structure encapsulated in PLGA/PEO polymeric matrix. MTT assay revealed the strong cytotoxic activity of FA-encapsulated PLGA/PEO nanofibers against MCF-7 cell line, which was primarily due to the initiation of apoptosis in tumor cell. Furthermore, these FA-encapsulated PLGA/PEO nanofibers demonstrated cytocompatibility when tested on embryonic kidney (HEK-293) cells. The findings suggested that the incorporation of FA in nanofibers may reduce the chemotherapeutic side effects and can be useful in providing a high local drug concentration to destroy the tumor cells. Therefore, these fabricated electrospun nanofibers are viewed as future candidates for antitumor drug delivery as well as potential local chemotherapeutic agent for breast tumor formations.

## Electronic supplementary material

NMR analysis data for native FA, PLGA/PEO nanofibers and FA-encapsulated PLGA/PEO nanofibers confirming the stability of FA after encapsulation has been provided as supplementary information.

Below is the link to the electronic supplementary material.

Supplementary material 1 (TIFF 348 kb)
