# Supplementary figures and images for: Antiproliferative activity of ferulic acid-encapsulated electrospun PLGA/PEO nanofibers against MCF-7 human breast carcinoma cells
Source: 3 Biotech. 2014 Jun 19;5(3):303–15. doi: 10.1007/s13205-014-0229-6 (PMC4434418; doi:10.1007/s13205-014-0229-6)

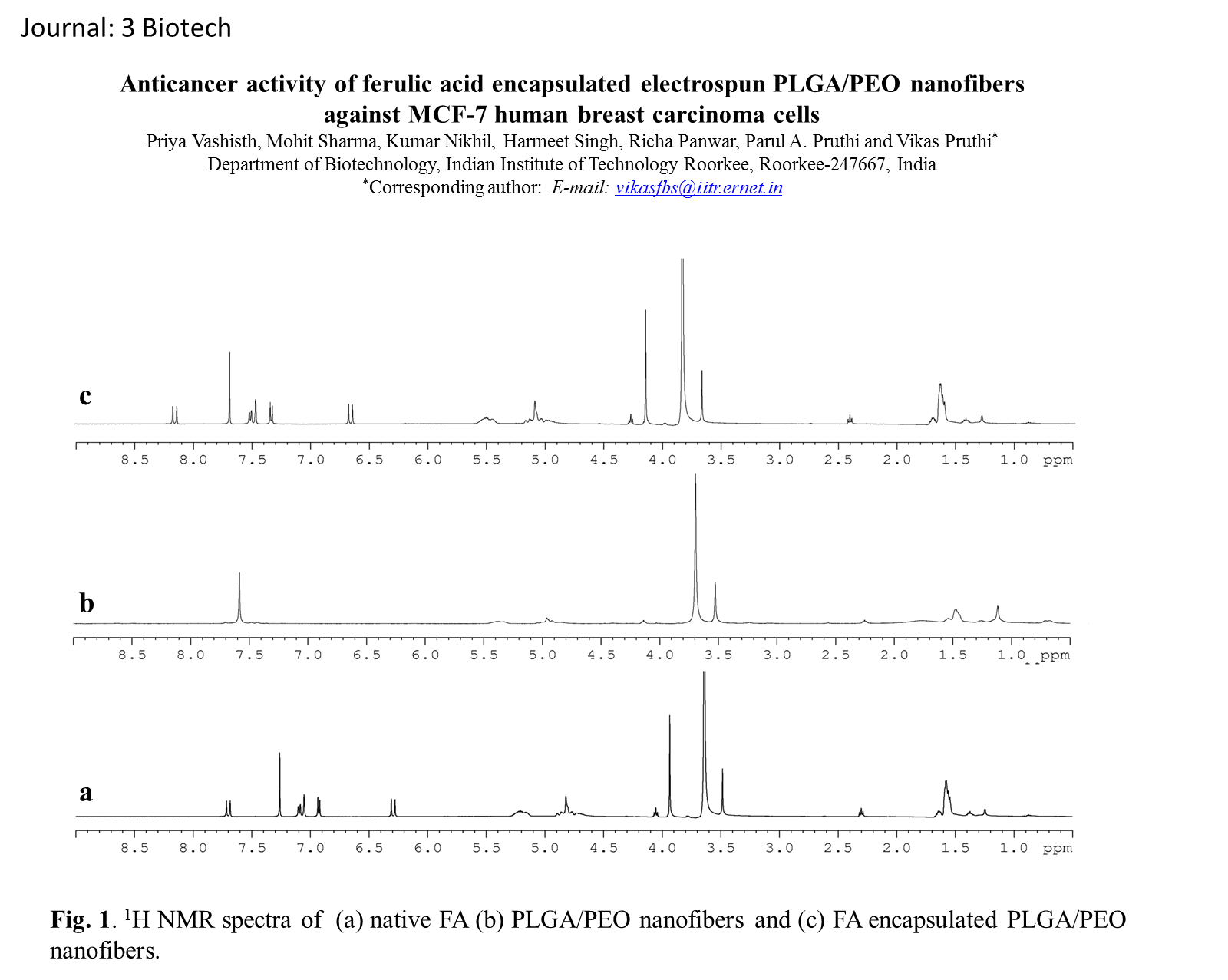

Supplement: Supplementary file 1 — Supplementary material 1 (TIFF 348 kb) [file 13205_2014_229_MOESM1_ESM.tif]
